# Supplementary material for: Emulation of epidemics via Bluetooth-based virtual safe virus spread: Experimental setup, software, and data
Source: PLOS Digit Health. 2022 Dec 2;1(12):e0000142. doi: 10.1371/journal.pdig.0000142 (PMC9931351; doi:10.1371/journal.pdig.0000142)
Supplement: S1 Appendix — Details of Prize draw rules. (PDF) [file pdig.0000142.s001.pdf]

Appendix 1: Prize draw rules

As participants run the Safe Blues app within the geofence area (See Fig 5), they collect hours which increase their chance of winning prizes. This is done by accumulating *campus hours* which are then translated into *eligible hours* via the rules described below. During Phase 1 we used a simpler set of rules, and introduced the 'invite a friend' mechanism from Phase 2 onward intending to increase participant numbers. See also the prizes web page, `https://safeblues.org/prizes/`.

This is an overview of the rules:

- For every hour running the app on campus, one *campus hour* is collected.
- Campus hours are accumulated since joining, or since the start of the current phase (campus hours are not collected during *paused periods* (see Table 1).
- A participant may collect up to 200 campus hours per phase.
- A maximum of 10 campus hours per day can be collected.
- Campus hours are converted to *eligible hours* as follows:
  - Each of the first 20 campus hours counts for 2 eligible hours.
  - After the first 20 campus hours, each additional campus hour counts as 1 eligible hour.
- Participants may obtain more eligible hours via the 'invite a friend' mechanism.
  - After accumulating 20 campus hours, participants may invite up to 10 friends to join the experiment and thereby receive bonus eligible hours.
  - For every friend they invite, after the friend collected 20 campus hours, the inviting participant receives 5 additional eligible hours.
  - The mechanism for inviting a friend is by generating a 6 digit invite code and asking the friend to enter that code.
  - Invited friends will receive 5 eligible hours when they sign up.
- Each phase with the exception of phase 3, has a prize draw at the end of the phase. Additionally, in place of the phase 3 prize draw, which was voided due to lockdown, there is a special prize draw (see below)
- In each prize draw (for phases 1, 2, 4, and 5), prizes are drawn as follows:
  - The chance of winning a prize is based on the eligible hours divided by the total number of eligible hours of all participants.
  - There are 9 prizes in each draw. A single top prize (iPad Pro), 3 second-tier prizes (Android mobile phones), and 5 third-tier prizes (FitBit tracker).
  - A participant may win at most one prize in a draw and this works as follows. First, everyone competes for the top prize and the winner is removed from the pool. Then all remaining participants compete for the second-tier prizes, each time removing the winner. Similarly for the third-tier.
  - A participant is eligible to win at most a single prize from each tier over the course of the experiment. For instance, a winner of the top prize in the first draw will be excluded from winning the top prize in draws that follow (but may still win other prizes).
- Participants can track both their collected campus hours and eligible hours per phase and see how many hours they have collected relative to the distribution of hours collected by other participants (see Fig 3, bottom left plot for a snapshot of the participants leader board showing this distribution).
- All participants are emailed a full report from the prize draw and winners collect directly from experiment staff.

- Winners are asked if they wish to have their picture and short bio posted. This is optional.
- There is a special prize draw in place of phase 3 during the period between phase 3 and phase 4. In this prize draw 10 participants have a chance to win a Fitbit tracker. The prize draw is performed by uniformly selecting 10 winners among all those participants who have accumulated 5 or more campus hours during the period of phase 2.
