# Supplementary material for: Emulation of epidemics via Bluetooth-based virtual safe virus spread: Experimental setup, software, and data
Source: PLOS Digit Health. 2022 Dec 2;1(12):e0000142. doi: 10.1371/journal.pdig.0000142 (PMC9931351; doi:10.1371/journal.pdig.0000142)
Supplement: S2 Appendix — Details of the Safe Blues app software. (PDF) [file pdig.0000142.s002.pdf]

# Appendix 2: App software

The Safe Blues Android app is based on the Trace Together Android App (Open Trace) [1] and was forked from the Open Trace Android version in April, 2020. The Open Trace software is designed for contact tracing, however we modified it to support the Safe Blues protocols. The app is written in the Kotlin language and has been made available on Google Play since March 2021.

The app is now specifically tailored for the Safe Blues campus experiment and includes on–boarding screens that assign the app instance a unique 10 digit ID. There is no email or other authentication information queried in the app. With the exception of the basic on–boarding screens, there is no user engagement in the app as it runs in the background. The app requires the user to enable location services and Bluetooth. Bluetooth is clearly needed for strand propagation. Location services are not directly part of the Safe Blues system but are needed to recognize that participants are in the geofenced area for the purpose of allocating prizes.

The initial phase of the experiment (see Table 1 in Appendix 6) included the release of several versions of the app that were automatically updated by participants. The sequence of these versions fixed several initial bugs. The most notable bug was a non-random initialization of the random number generator in the app, which caused all participating phones to seed Safe Blues strands in unison. Specifically, in strand batch `1.01` all participating phones decided together whether to seed a strand or not. Once this bug was fixed, a new app version was deployed. This deployment process included the strand batch `1.02` which did not include any strands per-se.

Due to limitations on control over the underlying Bluetooth hardware, the app transfers information between two phones by pairing them together for a brief period of time. We call each such Bluetooth interaction a “ping”. Each time two phones ping each other, they transmit their set of infectious strands as well as the strength of their transmitter. While the app is running, it continuously tries to ping other phones that are part of the experiment, including phones that have been pinged recently. These pings are then combined together into longer Safe Blues sessions (capped at 30 minutes) after which the Safe Blues simulation step runs. The original rationale for the session concept was to allow more flexibility in setting the infection mechanics of the simulation: our system could, for instance, be used to investigate the effect of a perfect contact tracing regime in tandem with these virtual pandemics (where a contact is traced if they spend a minimum duration at a minimum distance with a contact). Furthermore, the underlying Bluetooth systems are far from ideal for this, exhibiting congestion in large groups of participants and being unreliable even when there are few participants. The session system accommodates for some of this unreliability while also giving more accurate estimates of distance.

## References


[1] BlueTrace. OpenTrace; 2020 April [cited 21 December 2021]. Available from: `https://github.com/opentrace-community`.
