# Supplementary material for: Emulation of epidemics via Bluetooth-based virtual safe virus spread: Experimental setup, software, and data
Source: PLOS Digit Health. 2022 Dec 2;1(12):e0000142. doi: 10.1371/journal.pdig.0000142 (PMC9931351; doi:10.1371/journal.pdig.0000142)
Supplement: S3 Appendix — The ADS & PMS technical details. (PDF) [file pdig.0000142.s003.pdf]

Appendix 3: ADS and PMS servers technical details

The Safe Blues experiment system separates the anonymous data server (ADS) and the participant management system (PMS). The ADS is part of the operational Safe Blues system and is used to push new strands to the client apps and record phone strand information through anonymized client IDs generated within the app. The PMS is a system designed specifically for the experiment and is primarily aimed at participant management and prizes. It records how long the app was running in the background while the participant was within the experiment geofence. The PMS accomplishes this by using a separate "experiment ID" that is Completely isolated from the client ID in the ADS. Both systems run in parallel on the phone: the ADS is part of the experiment, while the PMS simply checks whether the participant is on campus with the required permissions and Bluetooth turned on, and relays summary counts to the PMS server.

We chose to physically separate these two servers in order to provide an extra layer of privacy protection for participants as well as to enable future Safe Blues deployments (perhaps operational) to use the ADS while not the PMS. In this separation, the PMS is hosted on a Nectar Research Cloud server managed by The University of Auckland, whilst the ADS is hosted on the commercial Amazon Web Services (AWS) Cloud.

The ADS uses a PostgreSQL database and exposes a gRPC API for phones indicating strand (and virtual social distancing) information, as well as a RESTful admin API. Phones running the app send messages to the ADS every 15 minutes, informing it of the status of the strands. The following are the protocol buffer messages about strands:

```
message Strand {
    string name = 13;
    int64 strand_id = 1;
    google.protobuf.Timestamp start_time = 2;
    google.protobuf.Timestamp end_time = 3;
    double seeding_probability = 4;
    // the two parameters of the infection probability map
    double infection_probability_map_p = 5; // strength
    double infection_probability_map_k = 6; // radius
    double infection_probability_map_l = 7; // unused
    // mean and shape of the gamma distribution for incubation period
    double incubation_period_mean_sec = 8;
    double incubation_period_shape = 9;
    // mean and shape of the gamma distribution for infectious period
    double infectious_period_mean_sec = 10;
    double infectious_period_shape = 11;

    uint32 minimum_app_version = 12;
}
```

Messages from phones to the ADS are encoded as follows:

```
message InfectionReport {
    string client_id = 1;
    int32 version_code = 5;

    repeated int64 current_incubating_strands = 2;
    repeated int64 current_infected_strands = 3;
    repeated int64 current_removed_strands = 4;
}
```

All such incoming messages are stored on the ADS Postgres database. Phones are only identified by a temporary 256-bit client ID that changes every 24 hours. Appendix 4 describes the algorithm running on the ADS for interpolation and imputation. Note that the ADS is not aware of the 10 digit participant ID which was only created for the purposes of the experiment.

The PMS stores a list of email addresses associated with each participant ID but does not store any further personal participant information (that is, we do not keep home addresses, names, afflictions, or other private information). The PMS receives messages from the phones via a restful JSON API indicating time spent on campus. An example of such a message is below (where `duration` and `count_active` are in units of 15 minute intervals, and the `truncated_entry_time` is the UNIX timestamp of the entry into campus. As can be seen, these messages indicate the time on campus. The phone generates such a message whenever leaving the geofenced area.


```
{
    "participant_id": 1234567890,
    "version_code": 60,
    "statuses": [
        {
            "status_id": 112,
            "truncated_entry_time": 18731,
            "duration": 12,
            "count_active": 11
        },
        {
            "status_id": 113,
            "truncated_entry_time": 18944,
            "duration": 17,
            "count_active": 17
        },
        ...
    ]
}
```


As shown in the bottom left image of Fig 3, the PMS aggregates these messages in a MySQL database, which is then queried for prize information and for presenting users with their current leader-board standings. The PMS also acts as a web server for a React-based website used for experiment registration, the 'invite a friend' mechanism, and the aforementioned leader-board.

Some specific PMS information is made available in accordance with the ethics approval. This is the total number of participants on campus and the total number of registered participants, as shown in Fig 6 (left plot), as well as summary of the distribution of daily campus hours of participants using the mean and a 5-number summary (see the right plot in Fig 6).
