# Supplementary material for: Emulation of epidemics via Bluetooth-based virtual safe virus spread: Experimental setup, software, and data
Source: PLOS Digit Health. 2022 Dec 2;1(12):e0000142. doi: 10.1371/journal.pdig.0000142 (PMC9931351; doi:10.1371/journal.pdig.0000142)
Supplement: S4 Appendix — Details of the interpolation and imputation algorithms. (PDF) [file pdig.0000142.s004.pdf]

Appendix 4: Interpolation and imputation algorithm

The epidemiological status of strands is stored in the ADS as a collection of reports received from participants' smartphones. The interpolation and imputation problem is to use these reports to reconstruct each participant's infection status at arbitrary times throughout the entire day. Specifically, the way in which the experimental data is collected and stored results in two issues that must be resolved: (a) a participant's status before any reports were received and (b) a participant's status during the time between reports.

Here, in describing the method used to solve the aforementioned problems, we fix a 24-hour UTC interval, an active strand, and a 256-bit ID. Note that, because a participant's anonymity 256-bit ID changes every 24 hours, our procedure cannot make use of any information before this window. We collect from the ADS database the following information: the time at which the participant sends their first report, T_F; the time at which the participant first reported a state of "exposed", T_E; the time at which the participant first reported a state of "infectious", T_I; and the time at which the participant first reported a state of "recovered", T_R. We choose $T_R = \infty$ if a "recovered" report is never received, $T_I = T_R$ if an "infectious" report is never received, and $T_E = T_I$ if an "exposed" report is never received. Moreover, given any $T \in \{T_E, T_I, T_R\}$ with $T = T_F$, we set $T = 0$. The participant's state at an arbitrary time t during the day is given by

$$\begin{cases} \text{“susceptible”}, & t < T_E, \\ \text{“expected”}, & T_E \leq t < T_I, \\ \text{“infectious”}, & T_I \leq t < T_R, \\ \text{“recovered”}, & T_R \leq t. \end{cases} \tag{1}$$
