# Supplementary material for: Emulation of epidemics via Bluetooth-based virtual safe virus spread: Experimental setup, software, and data
Source: PLOS Digit Health. 2022 Dec 2;1(12):e0000142. doi: 10.1371/journal.pdig.0000142 (PMC9931351; doi:10.1371/journal.pdig.0000142)
Supplement: S5 Appendix — Details of strands and their purposes. (PDF) [file pdig.0000142.s005.pdf]

Appendix 5: Strand details

Here we overview the main purpose of the batches of experiment strands at different stages of the experiment. Specific strands in each batch can be identified from the `strand_id` parameter listed in the `strand.csv` file in the Safe Blues Experiment Data repository (see also Appendix 6). Table 1 details the total number of strands released for each batch during phases 1–3 of the experiment, as well as the strand IDs associated with them.

Phase	Batch	No. of strands	Strand IDs	Type	Main Purpose
0	`0.01`	50	1–50	SEIR, SIR, SEI	The `debug` version, which is the first test of system in a workshop involving 10 users.
1	`1.01`	162	51–212	SEIR, SEI, SI	First time testing the system on the experiment population. A bug ("same seed") was detected.
1	`1.02`	1	213	SI	To test the system after fixing the "same seed" bug.
1	`1.03`	10	214–223	SEI	After the "same seed" bug, this was an intermediate debug batch while waiting for Google Play app update.
1	`1.04`	162	224–385	SEIR, SEI, SI	Same structure of strands as `1.01` released after fixing the "same seed" bug.
1	`1.05`	30	386–415	SI	Experimentation of the effect of strength and maximal infection distance on infection via SI strands.
1	`1.06`	90	416–505	SI	Further experimentation of the effect of maximal infection distance on infection via SI strands.
1	`1.07`	60	506–565	SI	Testing the virtual social distancing feature.
2	`2.01`	600	566-1165	SEIR, SIR, SEI, SI	Main release of all types of strands to begin Phase 2 of the experiment. These strands were planned to live until the end of Phase 3.
2	`2.02` – `2.06`	600 (per batch)	1166–4023	SEIR, SIR, SEI, SI	Weekly releases identical to `2.01` during the first month of the lockdown.
3	`3.01` – `3.22`	6 (per batch)	4024–4155	SI	As Auckland is still in lockdown, releasing 6 strands per week until the planned start of Phase 4 in case of any changes in behavior over this period
4 & 5	-	-	-	-	Strands for phases 4 and 5 will be similar to 2.01, currently planned with a release of new strands every two weeks during those phases.

Table 1: Batches of strands and their main purpose in the campus experiment.

We now overview the batches of Table 1 starting with the `debug` batch, `0.01`, and up to the Phase 3 final batch, `3.22`. Batch `0.01` or the `debug` batch was used as an initial test of the app prior to deploying it to the experimental population. This test trial involved 10 people who attended a Julia language meetup at The University of Queensland on April 21, 2021. This batch contains 50 strands in total, with a combination of SEIR, SIR, and SEI types. Following this, batch `1.01` was the first experimental test of the system as part of The University of Auckland experiment. Immediately after release of this batch, we observed that all participating

devices were initialized with the same random number generator seed. This bug, named as the "same seed", caused all phones to make the same decision about seeding a given strand or not. The bug was fixed and a single SI type strand was released as batch `1.02` to test this. While waiting for a Google Play update for the new app version, strands from batch `1.03` were released as an intermediate step for further testing. These were 10 SEI type strands. Once the bulk of the experiment participants updated their app to the new version, batch `1.04` was released with the same composition of strands initially intended in `1.01`. This batch, `1.04`, constitutes the main experimental batch within Phase 1.

The remaining batches of Phase 1, `1.05 – 1.07`, were aimed at further calibration of the strength and maximal infection distance parameters (as described in Eq (2)), as well as testing the virtual social distancing feature (see Materials and methods section in the main document). Specifically, `1.05` tested a range of distance and strength parameters and their effects on virtual viral spread, and `1.06` refined the search grid in the maximal distance parameters. We were able to see a clear effect of the maximal infection distance parameter on viral spread in both cases (see Fig 9). However, we were unable to detect a significant effect of the strength parameter within this parameter search. We released strands in batch `1.07` at the beginning of the week (Monday morning New Zealand time), and by Wednesday we had inflicted various levels of virtual social distancing, with factors ranging from 1.25 to 3.0. Measurements from this batch clearly indicated that the virtual social distancing mechanism has a significant impact on strand transmission (see Fig 9).

Moving onto the batches of phases 2 and Phase 3, the major batch providing data to date is `2.01`. This batch was released one week into Phase 2 (on Thursday, July 29) and included an extensive variety of strand types based on experience gained in Phase 1. Three weeks after the release, the major New Zealand lockdown took place and this immediately affected strand propagation (see Fig 10 presenting all strands from this batch). With anticipation of the lockdown potentially lifting, we released batches `2.02 – 2.06` weekly where each such batch contains the same types of strands as `2.01`. These batches have not yielded meaningful infections due to the continued lockdown. Finally, throughout Phase 3, and the interim period between Phase 3 and Phase 4, we released the weekly batches `3.01 – 3.22`. These batches, each with only 6 SI strands, are intended to "keep alive" the Safe Blues system.
