# Supplementary material for: Emulation of epidemics via Bluetooth-based virtual safe virus spread: Experimental setup, software, and data
Source: PLOS Digit Health. 2022 Dec 2;1(12):e0000142. doi: 10.1371/journal.pdig.0000142 (PMC9931351; doi:10.1371/journal.pdig.0000142)
Supplement: S6 Appendix — Structure of Safe Blues dataset. (PDF) [file pdig.0000142.s006.pdf]

Appendix 6: Data structure

The Safe Blues dataset is organized in the `data` repository as shown in Fig 1. The strand parameters determine the epidemiological behavior of each virtual virus–like token, including its transmissibility, incubation duration, and infection duration. A table of these parameters is stored in `data/strands.csv`. This table contains a row for each strand circulated during the campus experiment and contains columns described in Table 1. The transmission data provides aggregate measurements of the spread of strands throughout their

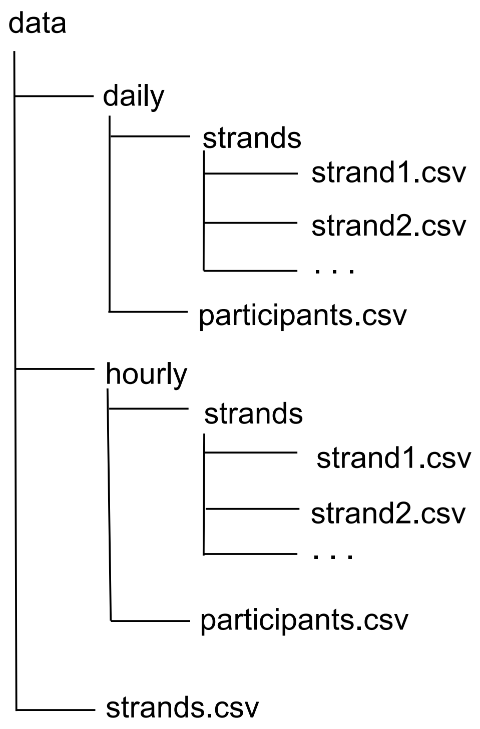

Figure 1: Safe Blues Data Structure.

reporting periods. We store the progression of the $\mathtt{i}^{\mathrm{th}}$ strand (`strand_id = i`) as a daily and hourly time series in `strand(i).csv` file. These tables have a row for each time point (either daily or hourly) and have the columns described in Table 2. The participant data presents aggregate information on the engagement of participants throughout the course of the experiment. This is also available as either a daily or hourly time series in `participants.csv` files. Again, these tables contain a row for each time point (either daily or hourly) and contain the columns described in Table 3.

Name	Description
strand_id	The unique numerical identifier given to each strand
batch	The release batch or group of strands within which each strand
model	The epidemiological model used by each strand (either SI, SEI, SIR, or SEIR)
start_utc	The time at which each strand's reporting begins in UTC timezone
start_nzt	The time at which each strand's reporting begins in NZST/NZDT timezone
seed_utc	The time at which each strand's initial infections occur in UTC timezone
seed_nzt	The time at which each strand's initial infections occur in NZST/NZDT timezone
stop_utc	The time at which each strand's reporting ends in UTC timezone
stop_nzt	The time at which each strand's reporting ends in NZST/NZDT timezone
initial	A participant's probability of initial infection for each strand
strength	The strength of transmission for each strand
radius	The maximal infection distance of transmission for each strand
incubation_mean	The mean parameter for each strand's gamma distributed incubation duration
incubation_shape	The shape parameter for each strand's gamma distributed incubation duration
infection_mean	The mean parameters of each strand's gamma distributed infection duration
infection_shape	The shape parameters of each strand's gamma distributed infection duration

Table 1: Description of the columns in strands.csv. The incubation_mean and incubation_shape values are missing when the model is either SI or SIR type. The infection_mean and infection_shape values are missing when the model is either SI or SEI type.

Name	Description
strand_id	The unique numerical identifier given to each strand
time_utc	The time of each measurement in UTC timezone
time_nzt	The time of each measurement in NZST/NZDT timezone
susceptible	The current number of participants who are susceptible to the strand
exposed	The current number of participants who are incubating the strand
infected	The current number of participants who are infected with the strand
recovered	The current number of participants who have recovered from the strand
distance_factor	The artificial distance multiplier used to emulate social distancing

Table 2: Description of the columns in the strand_i.csv file, where i is the ith strand. The values for exposed column are missing when the model is either SI or SIR type. The values for recovered column are missing when the model is either SI or SEI type.

Name	Description
time_utc	The time of each measurement in UTC timezone
time_nzt	The time of each measurement in NZST/NZDT timezone
count_campus	The number of participants on campus during the current day
count_reporting	The number of participants whose phones are sending reports during the current UTC day
count_registered	The number of participants who have registered for the experiment by the current day
hours_mean	The mean number of campus hours
hours_min	The minimum number of campus hours collected
hours_q1	The lower quartile number of campus hours collected
hours_q2	The median number of campus hours collected
hours_q3	The upper quartile number of campus hours collected
hours_max	The maximum number of campus hours collected

Table 3: Description of the columns in the participants.csv files. All statistics reported in the two participants.csv files are in reference to NZST/NZDT days except for count_reporting, which is in reference to UTC days. The mean and 5 number summary correspond to the campus hours collected by participants who attended campus on the current day. Values for the mean and 5 number summary are missing when count_campus is less than or equal to 5.
